# Expression of Concern: siRNA-mediated knockdown against NUF2 suppresses pancreatic cancer proliferation *in vitro* and *in vivo*

**DOI:** 10.1042/BSR-20140124_EOC

**Published:** 2021-04-19

**Keywords:** NUF2, pancreatic cancer, RNA interface, tumourigenesis, treatment

The Editorial Office has been made aware of potential issues surrounding the scientific validity of this paper, hence has issued an expression of concern to notify readers whilst the Editorial Office investigates.

